# A double-edged sword: parental care increases risk of offspring infection by a maternally vectored parasite

**DOI:** 10.1098/rsbl.2022.0007

**Published:** 2022-06-01

**Authors:** Rebecca Jean A. Millena, Jay A. Rosenheim

**Affiliations:** ^1^ RGGS, Invertebrate Zoology, American Museum of Natural History, New York, NY, USA; ^2^ Ecology and Evolution, Entomology and Nematology, University of California Davis, Davis, CA, USA; ^3^ Department of Entomology and Nematology, and Center for Population Biology, University of California Davis, Davis, CA, USA

**Keywords:** parental care, vertical transmission, *Ammophila*, Strepsiptera, *Paraxenos lugubris*, provisioning

## Abstract

Parental care can protect offspring from predators but can also create opportunities for parents to vector parasites to their offspring. We hypothesized that the risk of infection by maternally vectored parasites would increase with the frequency of mother–offspring contact. *Ammophila* spp. wasps (Hymenoptera: Sphecidae) build nests in which they rear a single offspring. *Ammophila* species exhibit varied offspring provisioning behaviours: some species enter the nest once to provision a single, large caterpillar, whereas others enter the nest repeatedly to provision with many smaller caterpillars. We hypothesized that each nest visit increases the risk of offspring parasitism by *Paraxenos lugubris* (Strepsiptera: Xenidae), whose infectious stages ride on the mother wasp (phoresy) to reach the vulnerable *Ammophila* offspring. We quantified parasitism risk by external examination of museum-curated *Ammophila* specimens—the anterior portion of *P. lugubris* protrudes between the adult host's abdominal sclerites and reflects infection during the larval stage. As predicted, *Ammophila* species that receive larger numbers of provisions incur greater risks of parasitism, with nest provisioning behaviour explaining *ca* 90% of the interspecific variation in mean parasitism. These findings demonstrate that parental care can augment, rather than reduce, the risk of parasite transmission to offspring.

## 1. Introduction

Parental care can provide important fitness benefits to offspring. By protecting offspring against natural enemies, including predators, pathogens and parasites, parents can substantially enhance the likelihood of successful offspring development [1,2]. Parental care is, however, associated with costs for the parents. Several studies have demonstrated that the costs of extended parental care can include enhanced risks of predation or parasitism for the parents [2–5].

Although it is generally expected that the offspring benefit from parental care, in some cases, parental activity associated with the care of offspring can also attract the attention of predators. Studies of nesting birds have demonstrated increased predation risk incurred by offspring with increased parental care [6]. Here we aim to examine another potential cost of parental care that may be incurred by offspring: enhanced risk of parasitism. We conduct what is, to our knowledge, the first cross-species comparison of the influence of parental care strategies on offspring parasitism risk.

Some parasites have exploited parental care to create opportunities for transmission, thereby converting the expected fitness benefits for offspring into fitness costs. This can be important either when the parent is itself infected and thus can be a source of infectious propagules (vertical transmission), or when the parent is uninfected but can still act as a vector of phoretic infectious stages of a parasite [7,8].

We examined the relationship between parental care and parasitism risk by studying solitary ground-nesting wasps in the genus *Ammophila* (Hymenoptera: Sphecidae) and their parasite *Paraxenos lugubris* (Strepsiptera: Xenidae) [9–11]. *Paraxenos* is an endoparasite that causes parasitic castration of its hosts—parasitized female *Ammophila* adults do not exhibit normal nesting behaviour, instead spending all of their time visiting flowers to imbibe nectar. When an *Ammophila* harbouring a mature female *Paraxenos* visits a flower, the *Paraxenos* female releases her free-living, phoretic first instar ‘triungulin’ larvae onto the flower [9,12]. This infective stage cannot directly infect adult hosts; rather, it must gain access to the vulnerable, larval stage of its host, *Ammophila*. The triungulin larvae wait on the flower for an adult *Ammophila* to visit, upon which they will attach to the wasp. If the unsuspecting wasp proves to be a female, she may then carry the parasites to her nest, where they disembark and attack her developing offspring. Thus, the mother functions as a vector of the infective stage of *P. lugubris* [9]. Because the nests of wasps and bees are typically highly protected, many parasites employ phoresy to gain entry, including satellite flies (Diptera: Miltogrammini), meloid and rhipiphorid beetles, parasitic mites, and fungal pathogens [13–16].

*Ammophila* wasps dig single-celled nests in which they rear a single offspring, providing it with caterpillars as food. *Ammophila* spp. have highly varied offspring provisioning behaviours: some *Ammophila* species provide a single, large caterpillar to each offspring, whereas other species provision each nest with multiple, small caterpillars [12,17,18]. Single-prey provisioning produces just one point of contact between the mother and offspring before the nest is sealed for the larva's development, whereas multiple-prey provisioning produces multiple points of contact [1,8]. Female *Ammophila* do clean the nest during their provisioning trips, and cleaning trips have been shown to remove some parasite larvae or adults from the nest [19,20]. We hypothesize that multiple contacts between the *Ammophila* mother and her offspring may still increase parasitism risk for the offspring if the increased parasite exposure more than offsets the increased nest cleaning activity associated with multiple provisioning visits. To test this hypothesis, we conducted a comparative test of parasitism risk versus nest provisioning behaviour for 16 species of *Ammophila* found in California.

## 2. Material and methods

**Figure 1.**
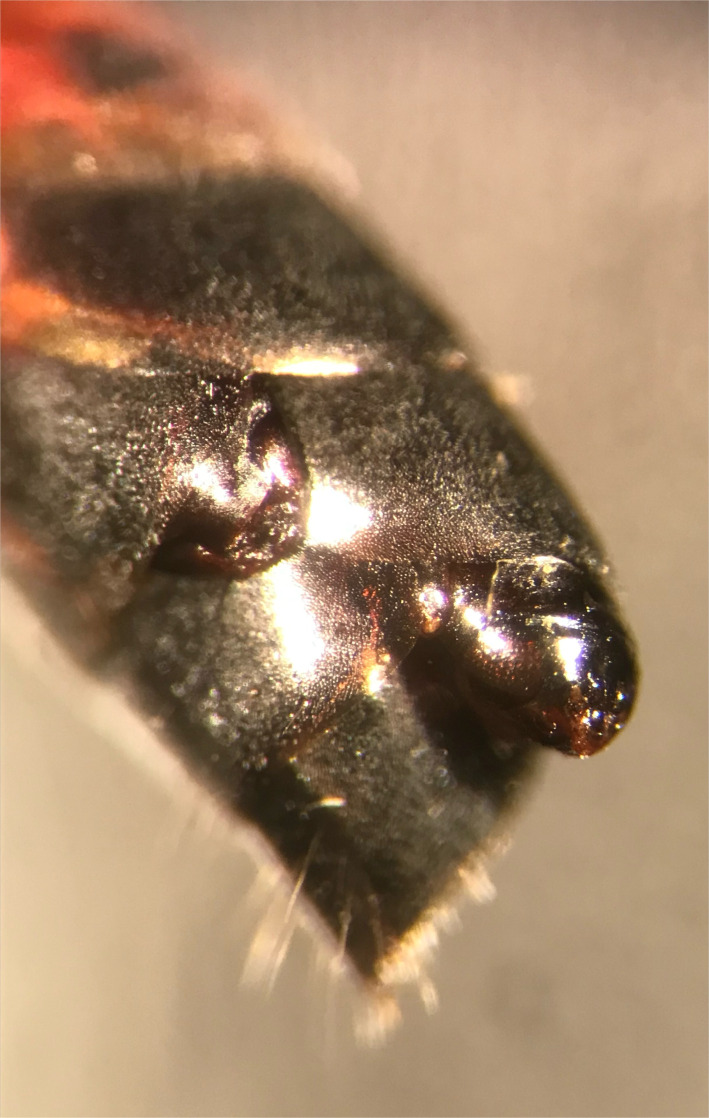
Two *Paraxenos lugubris* protruding between abdominal sclerites of *Ammophila azteca;* a female parasite is visible on the left and a male parasite in its pupal exoskeleton is visible on the right.

We gathered records of the provisioning behaviour for Californian species of *Ammophila* from the literature. Using ‘*Ammophila*’ and ‘provisioning’ as terms, we conducted searches via the Web of Science, BIOSIS and Google Scholar search engines. We supplemented the published literature with our own unpublished field observations. When multiple provisioning records existed for a species, we calculated an average across the studies to produce a single estimate for the mean prey provisioned. To quantify parasitism risk, we examined all specimens of *Ammophila* housed in the Bohart Museum of Entomology, University of California, Davis. This *Ammophila* collection had been curated by A. Menke, who described many of these species [21], producing a high degree of confidence in the species identifications of all specimens examined. Parasitized *Ammophila* specimens stored in the Strepsiptera collections were also included in our dataset. *Paraxenos lugubris* develops as an endoparasite, but its anterior end protrudes visibly from the abdomen of its host as it completes its development, allowing parasitism to be recorded in museum-preserved specimens (figure 1). This trait allowed us to convert an extensive museum collection of specimens into a large, comparative dataset quantifying parasitism. We examined each specimen with a stereomicroscope and scored the presence or absence of *Paraxenos*. Wasps were scored as parasitized when they had a female *Paraxenos* still present in their abdomen, or when they had a male *Paraxenos* either still present and enclosed in a pupal casing, or previously emerged from the abdomen, leaving behind a still-visible pupal exoskeleton (winged males emerge from their host to seek out females for mating, whereas the wingless females never leave their hosts). We also recorded *Ammophila* wing length as an index of host size, and the collection date for each specimen. Only species whose ranges include California were chosen for use in this study, as these species were reliably well represented in the museum's holdings. Our final dataset included all 16 species for which established estimates of mean prey provisioned were available (table 1). We scored a total of 8957 specimens collected between 1902 and 2009.

**Table 1.** Nest provisioning behaviours for each of 16 species of *Ammophila* whose ranges include California and for which estimates were available in the literature. Multiple provisioning records were averaged for eight species: *A. aberti*, *A. azteca*, *A. dysmica, A. harti, A. juncea, A. placida, A. pruinosa* and *A. urinaria*.

*Ammophila* species	mean prey provisioned per offspring	sources
*A. aberti*	8.5	Jiménez-Jiménez [22], Parker [1], Menke [23], Powell [24] and Evans [12]
*A. azteca*	7.5	Rosenheim (unpublished data), Bohart and Menke [25] and Menke [23]
*A. boharti*	1	Rosenheim (unpublished data)
*A. dysmica*	1.75	Rosenheim [19,26]
*A. femurrubra*	2	image and records taken by Melton in 2014, https://bugguide.net/node/view/1055016
*A. harti*	5	Hager & Kurzcewski [27], Parker [1], Menke [23] and Evans [12]
*A. juncea*	1.5	Parker [1], Menke [23] and Evans [12]
*A. marshi*	1	Rosenheim (unpublished data)
*A. nigricans*	1	Parker [1], Menke [23] and Evans [12]
*A. placida*	3	Menke [23] and Evans [12]
*A. procera*	1	Parker [1], Menke [23] and Evans [12]
*A. pruinosa*	7.5	Parker [1], Menke [23], Evans [12] and Hicks [28]
*A. stangei*	1	Rosenheim (unpublished data)
*A. urnaria*	3.5	Parker [1], Menke [23] and Evans [12]
*A. wrightii*	1	Parker [1] and Menke [23]
*A. zanthoptera*	1	Parker [1] and Evans [12]

We analysed parasitism data with a generalized linear mixed-effects model with binomial variance and a logistic link function, using the R package *lme4* [29] (electronic supplementary material, table S1). The model included the mean number of prey provisioned, month and size as fixed effects, species ID and collection location (county) as random effects, and parasitism (yes or no) as a response. The two continuous predictor variables, host wasp size and the mean number of caterpillars provisioned per nest, were not significantly correlated across species (*r* = 0.207, *N* = 16, *p* = 0.44). We included county in the model as a random effect to control for any possible spatial variation in parasitism rates. We also ran an ANOVA using the R package *car* [30] to evaluate the main effects of each variable in the model. To ensure the statistical independence of our species-level observations, we conducted phylogenetic contrasts on a subset of our data with the R package *ape* [31]. These analyses were based on a molecular phylogeny of the tribe Ammophilini published by Field *et al*. (2011), which includes 10 of the 16 *Ammophila* species in our main dataset: *A. azteca, A. aberti, A. dysmica, A. urnaria, A. femurrubra, A. nigricans, A. marshi, A. stangei, A. wrightii* and *A. procera* [17]. All statistical modelling code is available in the electronic supplementary material, appendix.

## 3. Results

**Figure 2.**
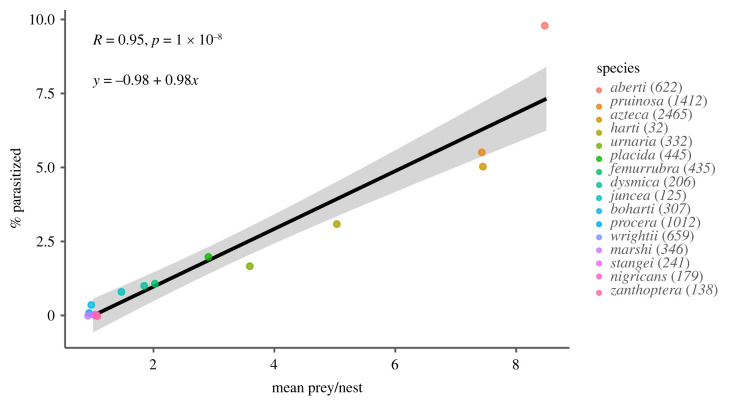
Parasitism data for 16 North American *Ammophila* species. Data points for singly provisioning species with zero parasitism were jittered along the *x*-axis for visibility. Shaded area represents the 95% confidence interval for the linear regression. Numbers in the legend indicate how many specimens were scored for each species.

The mean number of prey provisioned per offspring exerts a dominant influence on parasitism risk in *Ammophila* spp. (GLMM, effect for provision number: 0.425 ± 0.0390 (s.e.), *z* = 10.9, *p* < 0.0001; figure 2), explaining more than 90% of the observed interspecific variation in parasitism. Mean host size had no significant influence on observed parasitism (GLMM, effect for size: −0.055 ± 0.049, *z* = −0.650, *p* = 0.52). Seasonal variation in parasitism was observed (ANOVA, *p* < 0.0001), with lower parasitism rates observed in wasps collected in April, May and August when compared to January as a reference level (electronic supplementary material, table S1). Phylogenetic contrasts performed with the reduced dataset of 10 *Ammophila* species confirmed a significant effect on parasitism of the number of caterpillars provisioned per nest (1.064 ± 0.192, *z* = 0.000853, *p* < 0.0001; electronic supplementary material, figure S2).

## 4. Discussion

*Ammophila* species with more numerous nest provisioning visits incur an increased risk of parasitism by *Paraxenos lugubris*, a parasite that exploits the mother wasp as a vector for its triungulin larvae. Singly provisioning species had parasitism rates ranging from zero to 0.33%, whereas *Ammophila aberti*, with the highest mean number of caterpillars provisioned per offspring (8.5, table 1), had the highest parasitism rate (9.8%). Importantly, we can infer unambiguously that this positive correlation reflects an influence of wasp provisioning on parasitism risk, rather than an influence of parasitism on wasp nesting behaviour, because parasitized wasps are castrated by the parasite and do not nest. This host–parasite system reveals that parental care can be a double-edged sword—while *Ammophila* spp. mothers can often protect their offspring from parasites and predators, they can also act as vectors of the infectious stages of *Paraxenos*, elevating the parasitism risk for their offspring. Given that vertical transmission of pathogens is a major pathway of infection for parasites and pathogens [32–34], our results suggest that parasitism risk for offspring is likely to be an underappreciated cost of parental care.

It is possible that parasitized *Ammophila* specimens might have been under- or over-represented in the specimens saved by the museum. However, we think it is unlikely that any possible bias would have been connected to the parental care traits of the host species, which would have been unknown to the collectors in virtually all cases.

Five out of the seven singly provisioning species had no observed parasitism. This may reflect either that the true parasitism rate is too small to be detected in our samples, or that these species are not actually hosts for *P. lugubris*. Almost all of the species studied here were not previously documented as hosts for *Paraxenos* [10], and thus we cannot rely on the published literature to establish the full host range for this parasite. The basis for the extremely low parasitism rates observed for singly provisioning species is at present not fully explained. One way to quantify this deficit of expected parasitism for singly provisioning species is to note that the *y*-intercept of the linear regression shown in figure 2 is significantly negative, rather than being near zero, as would be expected if parasitism risk were directly proportional to the number of prey provisioned. Research exploring the cues used by the triungulin larvae of *Paraxenos* to disembark from their phoretic hosts may be needed to answer this question.

We similarly do not know the basis for the observed seasonal (monthly) variation in observed parasitism. There may be seasonal variation in the activity of *Paraxenos* that shapes these patterns; however, our data reveal only when the parasitized adults were collected, not when the parasites initially penetrated the host nest. It is difficult to connect the timing of the parasitism event with the timing of emergence of the parasitized adult, especially for multivoltine *Ammophila* species. Some helpful information has been published by Kathirithamby *et al.* [9] on the seasonal activity and life cycle of *P. lugubris* attacking *Ammophila*, but an explanation for the temporal variation in parasitism risk documented here requires further study [9].

This project has employed a comparative approach to test the hypothesis that parental care can amplify, rather than reduce, parasitism risk to offspring when parents can vector parasites to their offspring. Strepsipteran parasites may be especially valuable for comparative studies of host–parasite interactions, because data collection for a large number of host species over long time periods and with deep sample sizes for each host species is feasible. The insect specimens that have been curated in entomology museums preserve this record of parasitism. The ease of data collection will allow researchers to merge historical and contemporary datasets, providing more thorough analyses. This study joins many others demonstrating the key role of museums as repositories for key biological data [35–37].

## Data Availability

The data and code for this article are available from the Dryad Digital Repository: https://doi.org/10.5061/dryad.qnk98sfjg [38]. Metadata for the raw dataset are included as a separate tab in the raw data Excel file, and necessary information for the code (R scripts) is present as a README file. Provisioning and parasitism data; code for analyses: [38]. The data are provided in the electronic supplementary material [39].
